# Supplementary material for: Left Ventricular Mass Reduction by a Low-Sodium Diet in Treated Hypertensive Patients
Source: Nutrients. 2020 Nov 30;12(12):3714. doi: 10.3390/nu12123714 (PMC7761364; doi:10.3390/nu12123714)
Supplement: Supplementary file 1 [file nutrients-12-03714-s001.pdf]

Supplementary Table S1. Drug classes in the two subgroups from Time 0-1 to Time 2.

Drug Class	Reducers (N = 76)	Non Reducers (N = 62)
ACE/I	15	12
ARB	16	12
HTZ	2	2
CCB	5	3
ACE/I + HTZ	10	7
ARB + HTZ	10	9
(ACE/I or ARB) + CCB	15	15
(βB or CCB) + HTZ	3	2

Table S1: ACE/I (angiotensin converting enzyme inhibitors); ARB (angiotensin receptor blockers); HTZ (hydrochlorothiazide and thiazide diuretics); CCB (calcium channel blockers); βB (beta blockers). In the contingency analysis there were no significant differences between groups:

Chi^2: All drug classes df 0.7745, 7; *p*=.9977.

Chi^2: (ACE/I and ARB) vs others df 0.1103, 1; *p*=.7399.
(ACE/I or ARB)/CCB vs others df 0.0009, 1; *p*=.9760.
HTZ vs non-HTZ df 0.0007, 1; *p*=.9791.

Fischer: (ACE/I and ARB) vs others; *p*=.7995.
(ACE/I or ARB)/CCB vs others; *p*=1.000.
HTZ vs non-HTZ; *p*=1.000.

Supplementary Figure S1. LVH regression / LVH progression.

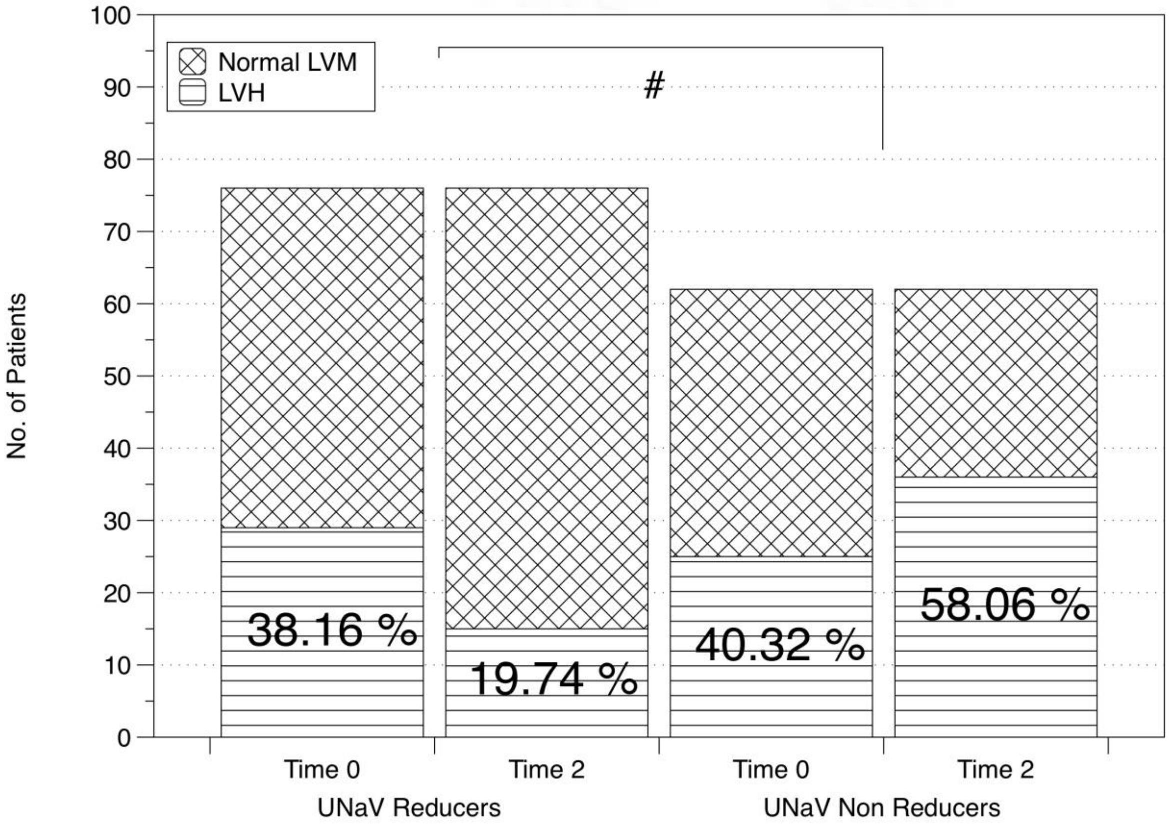

Figure S1: LVH regression in patients with sodium excretion reduction over time (UNaV Reducers), compared to LVH progression in patients without sodium reduction over time (UNaV Non Reducers). (Chi^2 test, # *p*=.002). Patients with LVH are expressed also as percent values (values inside bars). LVH: left ventricular hypertrophy; LVM: left ventricular mass; UNaV: 24-h urinary sodium excretion.
